# Maximal strength training-induced increase in efferent neural drive is not reflected in relative protein expression of SERCA

**DOI:** 10.1007/s00421-021-04807-0

**Published:** 2021-09-08

**Authors:** Tiril Tøien, Håvard Haglo, Stian Kwak Nyberg, Shalini Vasudev Rao, Astrid Kamilla Stunes, Mats Peder Mosti, Eivind Wang

**Affiliations:** 1 Department of Health and Social Sciences, Molde University College, Britvegen 2, 6410 Molde, Norway; 2 Myworkout, Medical Rehabilitation Clinic, Trondheim, Norway; 3 Department of Circulation and Medical Imaging, Faculty of Medicine and Health Sciences, Norwegian University of Science and Technology, Trondheim, Norway; 4 Cambridge Institute, University of Cambridge, Cambridge, UK; 5 Department of Clinical and Molecular Medicine, Faculty of Medicine and Health Sciences, Norwegian University of Science and Technology, Trondheim, Norway; 6 Medical Clinic, St. Olavs University Hospital, Trondheim, Norway; 7 Department of Medicine, University of Utah, Salt Lake City, UT USA; 8 Department of Østmarka, Division of Mental Health Care, St. Olavs Hospital, Trondheim University Hospital, Trondheim, Norway

**Keywords:** Strength training, SERCA, Efferent neural drive, Neural adaptations

## Abstract

**Introduction:**

Maximal strength training (MST), performed with heavy loads (~ 90% of one repetition maximum; 1RM) and few repetitions, yields large improvements in efferent neural drive, skeletal muscle force production, and skeletal muscle efficiency. However, it is elusive whether neural adaptations following such high intensity strength training may be accompanied by alterations in energy-demanding muscular factors.

**Methods:**

Sixteen healthy young males (24 ± 4 years) were randomized to MST 3 times per week for 8 weeks (*n* = 8), or a control group (CG; *n* = 8). Measurements included 1RM and rate of force development (RFD), and evoked potentials recordings (V-wave and H-reflex normalized to M-wave (M) in the soleus muscle) applied to assess efferent neural drive to maximally contracting skeletal muscle. Biopsies were obtained from vastus lateralis and analyzed by western blots and real-time PCR to investigate the relative protein expression and mRNA expression of Sarcoplasmic Reticulum Ca^2+^ ATPase (SERCA) 1 and SERCA2.

**Results:**

Significant improvements in 1RM (17 ± 9%; *p* < 0.001) and early (0–100 ms), late (0–200 ms) and maximal RFD (31–53%; *p* < 0.01) were observed after MST, accompanied by increased maximal *V*_max_/*M*_sup_-ratio (9 ± 14%; *p* = 0.046), with no change in H-reflex to M-wave ratio. No changes were observed in the CG. No pre- to post-training differences were found in mRNA or protein expressions of SERCA1 and SERCA2 in either group.

**Conclusion:**

MST increased efferent neural drive to maximally contracting skeletal muscle, causing improved force production. No change was observed in SERCA expression, indicating that responses to high intensity strength training may predominantly be governed by neural adaptations.

## Introduction

As a modality of heavy load strength training, maximal strength training (MST) is performed using loads of ~ 90% of maximal strength, few repetitions, and maximal mobilization of force in the concentric phase (i.e., maximal intended velocity). Compared to the more commonly used lower load training, which uses lower resistance (~ 65–75% of maximal strength) and typically 10–12 repetitions, MST increases maximal strength and rate of force development (RFD) almost twice as much (Heggelund et al. 2013). In addition to the beneficial effects on maximal muscle strength and RFD, MST is well-documented to reduce the energy cost of locomotion (Hoff et al. 1999, 2002; Storen et al. 2008) by improving skeletal muscle efficiency (Barrett-O’Keefe et al. 2012; Berg et al. 2018). However, the underlying neuromuscular adaptations responsible for the improved muscular performance are still unclear.

Since MST does not increase body mass in the first 8 weeks of training (Hoff et al. 2001; Heggelund et al. 2013; Wang et al. 2010), the main adaptations have been suggested to occur in the nervous system. Indeed, neural adaptations have been evident as an efferent neural drive enhancement, largely dependent on corticospinal factors, to maximally contracting skeletal muscle in the lower extremities (Toien et al. 2018a; Fimland et al. 2009). This efferent neural drive enhancement has been detected using evoked reflex recordings in the calf muscles, evident as an increased V-wave to M-wave (*V*_max_/*M*_sup_)-ratio, following leg press training. However, whether an increase in efferent neural drive may also be reflected in the musculature is uncertain. A key link between neural components and muscle contraction is the intramuscular calcium concentration [Ca^2+^] (Baylor and Hollingworth 2003). In short, an action potential leads to a passive release of Ca^2+^ from the sarcoplasmic reticulum (SR) causing increased myoplasmic [Ca^2+^], which in turn leads to a muscle contraction (Baylor and Hollingworth 2003). To terminate the muscle contraction there is an active Ca^2+^ reuptake to decrease [Ca^2+^] mainly via the SR Ca^2+^ ATPase (SERCA)-pumps (Baylor and Hollingworth 2003), making this process energy-dependent. Of particular interest in this excitation–contraction process, since MST improves skeletal muscle efficiency, muscular adaptations might contribute to alter energy-dependent processes. Thus, the relative expression of SERCA may change following MST, although it is unclear which effect, if any, training has on SERCA expression. Following 4 weeks of concurrent strength and endurance training, Skovgaard et al. (2014) observed decreased relative protein expression of SERCA1 (expressed in fast-twitch fibres), although this was not significant after 8 weeks, and no change in SERCA2 (expressed in slow twitch fibres) at either timepoint. However, concurrent interventions make difficult to distinguish the source of adaptation. In fact, similar results, i.e., decreased relative expression of SERCA1 and SERCA2, have been observed following long and short-term endurance training (Green et al. 2003, 2011; Majerczak et al. 2008), suggesting this may have been predominantly a result of the endurance intervention. Conversely, there appears to be an opposite effect of sprint training, where an increase in SERCA1 and SERCA2 expression has been documented (Ortenblad et al. 2000). Considering these uncertainties, the MST effects on relative SERCA expression should be examined in conjunction with the expected efferent neural drive adaptations.

The aim of the present study was, therefore, to examine the effect of leg press MST on relative SERCA expression in vastus lateralis along with efferent neural drive measurements, detected as *V*_max_/*M*_sup_-ratio. In accordance with previous literature, we hypothesised that MST would increase maximal strength, RFD and efferent neural drive, and that these enhancements would be accompanied by changed relative protein- and messenger RNA (mRNA) expression of SERCA1 and SERCA2.

## Methods

### Subject characteristics

A total of 16 young males participated in the study and were randomly assigned by a researcher not involved in the data collection and the training intervention to MST (*n* = 8, age: 24 ± 3 years, stature: 179 ± 5 cm) or a non-training control group (CG; *n* = 8, age: 24 ± 5 years, stature: 185 ± 8 cm). The number of participants in each group was based on previous literature (Green et al. 2003, 2011; Ortenblad et al. 2000). None of the subjects reported to engage in regular strength training of the lower extremities prior to participation in the study, and they had no known neuromuscular disease that could affect the study outcome. The study was approved by the local ethics committee and all subjects gave their written informed consent before taking part in any of the procedures. In the informed consent the participants were informed of their right to withdraw from the study at any time, but all subjects completed the testing and training. This project was performed according to the Declaration of Helsinki.

### Study timeline

Standardized test procedures were performed over two separate days at the same time of day, before and after the training period. On the first test day, the subjects started with a 10-min treadmill warm up followed by leg press maximal strength and RFD testing, and subsequent plantar flexion evoked reflex recordings. On the second test day (following 2–4 days of rest) vastus lateralis muscle biopsies were obtained from the subjects. Training was conducted three times per week for 8 weeks, and a minimum of 20 sessions had to be completed to proceed to the follow-up testing. There was a minimum of 2 days between the final training session and the follow-up test. No familiarization of the training or testing was performed, and the CG served as a time-control from pre-to post-test.

### Maximal strength and rate of force development

One repetition maximum (1RM) was obtained in a horizontal leg press (Technogym silver line, Italy) as a measure of maximal leg strength. After completing three warm up sets of eight, six and two repetitions at a light load, the load was gradually increased with 10–20 kg to reach 1RM within 5 lifts. When the participants were close to 1RM, determined by visual inspection of the participants’ effort, the increase in load for the next trial was no higher than 10 kg. The trials were separated by 3–4 min of rest. 1RM was defined as the highest load the participant was able to lift one time before failure. Each lift started with an eccentric phase (from near ~ 180° to ~ 90° angle in the knee joint), followed by a short stop (< 1 s) when the subjects’ knee angle was ~ 90°, before the concentric phase back to near ~ 180° in the knees. Careful consideration and instructions were given to avoid hyperextension of the knees. Range of motion of the knees was determined visually by a researcher with the aid of a goniometer. For visual representation of the 1RM and RFD test and training set up, please see previous studies from our laboratory (Hoff et al. 2007; Toien et al. 2018a). A recent systematic review concluded that intraclass correlation coefficient (ICC) for 1RM testing was good-to-excellent, with the median ICC at 0.97 and a low level of variation (< 10%) regardless of, e.g., familiarization sessions, experience with strength training and exercise selection (Grgic et al. 2020).

Following the termination of the 1RM test the subjects were given a 3–4-min rest period before performing the dynamic RFD trials in the same leg press apparatus. For these trials a standard weight of 70% of pre-test 1RM was used. The trial was performed on a force platform (9286AA, Kistler, Switzerland) sampling at 800 Hz. The platform was mounted on the leg press apparatus with a custom-built attachment and force was obtained via Bioware software v. 5.3.0.7 (Kistler, Switzerland). In each 1RM and RFD lift, the participant was instructed to perform the eccentric phase in a slow and controlled manner, before aiming to lift the weight as forcefully and fast as possible in the concentric phase. The subjects performed three RFD trials, of which the steepest force–time curve was used in further data analysis. RFD was calculated as Δforce/Δtime in the time intervals 0–30 ms, 0–50 ms, 0–100 ms, 0–150 ms, 0–200 ms, where 0 ms denotes the onset of concentric force production, along with maximal RFD in the steepest 10 ms of the force–time curve.

### Evoked reflex recordings

H-reflexes and V-waves were evoked in the tibial nerve located in the popliteal fossa of the right leg as an assessment of efferent neural drive to the lower extremities. The participants were seated in a custom-made isometric apparatus (Unhjem et al. 2016) with the ankle in a neutral position and the knee flexed at 90°. The skin was shaved, abraded (Nuprep, Weaver and company, Aurora, CO, USA) and thoroughly wiped clean with alcohol before placing self-adhesive pairs of bipolar Ag/AgCl electrodes (Ambu, M-00-S/50, Ballerup, Denmark) with an inter-electrode distance of 25 mm on the soleus muscle as recommended by SENIAM (Hermens et al. 2000) to record electric potentials. The skin-preparation procedure was included to ensure minimal resistance in the skin, and maximal interelectrode impedance level was set to 5 kΩ. Anatomical landmarks and measuring tape were used to identify the appropriate location of electrode placement, and pictures were taken of the relevant leg to ensure identical placement of the electrodes from pre- to post-test. A current stimulator (DS7AH, Digitimer, Welwyn Garden City, UK) gave a 1-ms square wave stimulus percutaneously to the tibial nerve in the popliteal fossa via hand-held, gel-coated (Lectron 2 conductive gel, Pharmaceutical Innovations Inc., Newark, NJ, USA) bipolar felt pad electrodes, which were 8 mm in diameter, and had 25 mm between the tips (Digitimer, Welwyn Garden City, UK). Electromyography (EMG) data were obtained with Megawin software (700,046 version 3.0), via the ME6000 Biomonitor (Mega Electronics LTD, Kuopio, Finland) at 2 kHz, common mode rejection ratio (CMRR) of 110 dB. The EMG-signals were amplified and band-pass filtered (8–500 Hz).

H-reflexes were obtained during 10% of isometric maximal voluntary contraction (MVC), to ensure stable motoneuron excitability and minimize postsynaptic effects (Knikou 2008). First, the optimal site for stimulation was located by placing the electrodes at the position evoking the largest H-reflex amplitude with concurrent lowest M-wave amplitude, before the current intensity was gradually increased by 2–5 mA searching for the maximal H-reflex peak-to-peak amplitude (*H*_max_). The stimulation intensity was carefully monitored to ensure similar M-wave responses during *H*_max_ between groups and from pre- to post-test [see Table 1; M at *H*_max_ (%*M*_max_)]. After *H*_max_ had been located the electrical stimulation current was increased further to elicit the maximal M-wave (*M*_max_) obtained during 10% MVC. When no further increase in M-wave amplitude was seen, despite increased electrical current, *M*_max_ was identified. To ensure a true *M*_max_ was reached, a supramaximal stimulus of 150% of the stimulus needed to evoke *M*_max_ was given (Aagaard et al. 2002b). *H*_max_ was normalized to *M*_max_. To minimize any conditional effects on H-reflexes, testing was performed in the same laboratory and by the same researcher at pre- and post-test.

**Table 1:** Absolute amplitudes (µV) and normalized evoked peak-to-peak amplitude potentials of the soleus muscle

	MST		CG	
	Pre	Post	Pre	Post
*H*_max_	3640 ± 2485	2780 ± 1498	2974 ± 927	2829 ± 995
*M*_max_	6037 ± 2540	5682 ± 2827	6995 ± 1922	6619 ± 1921
M at *H*_max_ (%*M*_max_)	23 ± 6	21 ± 7	23 ± 6	24 ± 8
*H*_max_/*M*_max_	0.57 ± 0.18	0.49 ± 0.07	0.45 ± 0.17	0.46 ± 0.19
*V*_max_	2363 ± 1626	2560 ± 1484	2661 ± 1626	2356 ± 1214
*M*_sup_	6292 ± 2635	6364 ± 2379	7018 ± 2464	6517 ± 2167
*V*_max_/*M*_sup_	0.36 ± 0.11	0.39 ± 0.14*	0.36 ± 0.11	0.34 ± 0.09

Data are presented as mean ± SD

*MST* maximal strength training, *CG* control group, *H*_*max*_ maximal H-reflex amplitude during 10% maximal voluntary contraction (MVC), *M*_*max*_ maximal M-wave amplitude during 10% MVC, *H*_*max*_*/M*_*max*_ maximal H-reflex amplitude/maximal M-wave amplitude, *V*_*max*_ maximal V-wave amplitude during MVC, *M*_*sup*_ maximal M-wave amplitude during maximal voluntary contraction, *V*_*max*_*/M*_*sup*_ maximal V-wave amplitude/maximal M-wave amplitude

*p ≤ 0.05 different from pre-test within group

Following the detection of *M*_max_, 6–8 V-wave recordings were performed. The participants were instructed to exert maximal, rapid voluntary muscle force, and V-wave responses were evoked by delivering a supramaximal stimulus to the tibial nerve during MVC. When a plateau of force was detected, the subjects were perceived to have reached MVC and the stimulus was applied. 1-min rest periods were given between each MVC. V-waves (*V*_max_) were normalized to the maximal M-waves obtained during MVC (*M*_sup_). The amplitude of the M-wave had to be ≥ 95% of *M*_sup_ and the force had to be ≥ 90% of MVC to be included in further analysis.

### Muscle biopsies

Muscle biopsies were taken 3.5 cm deep from the vastus lateralis, approximately 15 cm proximal to the knee and slightly distal to the ventral midline of the muscle (Richardson et al. 2000) following 2–4 days of rest after strength testing in both groups. Biopsies were taken at the same time of day for each subject, within 2 h of the pre-test biopsy. The subjects were asked to refrain from alcohol and strenuous exercise within 48 h leading up to the biopsy and stick to their normal eating habits on both occasions. A 6 mm Bergström needle attached to a suction syringe was used after injecting local xylocaine (1%) anaesthesia. At post-test the biopsies were randomly collected 1 cm distal or proximal to the pre-test biopsy. The biopsies were frozen in liquid nitrogen immediately after collection and stored in a − 80 °C freezer until further analyses.

### mRNA isolation and cDNA synthesis from muscle biopsies

Muscle samples (~ 30 mg) from the vastus lateralis were lysed in RNeasy lysis buffer using an electric knife-homogenizer, and total RNA was isolated using the RNeasy Fibrous Tissue Mini Kit (Qiagen, CA, USA). The amount of total RNA in each sample was measured using a NanoDrop Spectrophotometer, and equal amounts of total RNA from each sample were applied directly to obtain a first-strand complementary DNA (cDNA) using the iScript cDNA Synthesis Kit with oligo (dT) and random primers (Bio-Rad, CA, USA).

### Real-time PCR analyses

The real-time PCR analyses were carried out on a StepOne Real-Time PCR System with SYBR green dye for detection (Applied Biosystems, CA, USA) according to the protocol from SABiosciences. Primers were purchased from Biorad (NCBI). mRNA expression of the following genes were analyzed: Sarcoplasmic reticulum calcium ATPase 1, (Biorad ID, qHsaCID0017519), Sarcoplasmic reticulum calcium ATPase 2 (Biorad ID, qHsaCID0011088), and glyceraldehyde-3-phosphate dehydrogenase (GAPDH, Fwd: 5′TCTGACTTCAACAGCGACACC-3′; Rev: 5′-TGTTGCTGTAGCCAAATTCGT-3′). Data were calculated using the relative standard curve method, with GAPDH as the housekeeping gene.

### Western blot analyses

Proteins were extracted from the vastus lateralis muscle biopsies (∼30 mg) and lysed in NP40 buffer with 0.1 M DTT, protease inhibitor 1 and 2 (Sigma-Aldrich) and phosphatase inhibitors (Roche Diagnostics), using an electric knife-homogenizer. Protein extracts (10 μg protein/well) were separated on NuPAGE 4–12% Bis–Tris gels (Invitrogen) and electroblotted onto Immobilon PVDF membranes (Millipore). The membranes were blocked with 5% BSA in PBS-Tween and incubated overnight at 4 °C with monoclonal rabbit anti-SERCA1 ATPase antibody (ab129104, Abcam) 1:10 000, monoclonal mouse anti-SERCA2 ATPase antibody (MA3-919, Thermo-Scientific) 1:10 000 and monoclonal moues anti-GAPDH (ab9484, Abcam) 1:15 000, in 1% BSA in PBS-Tween. Secondary antibodies were HRP-conjugated rabbit anti-mouse IgG (P0260, Dako) 1:5000 or HRP-conjugated swine anti-rabbit IgG (PO399, Dako) 1:5000. Binding of antibodies was developed using SuperSignal West Femto Maximum Substrate (Thermo-Scientific) and visualized on LI-COR’s Odyssey-Mode imaging system. Protein expression was quantified using the ImageStudio version 3.1 software, and SERCA1 and SERCA2 expression were normalized to GAPDH expression.

### Training intervention

The training consisted of four sets of 4RM at an intensity of ~ 90% of 1RM in the leg press three times per week for 8 weeks. When a participant was able to lift a fifth repetition in a set, the load was increased by 5 kg in the next session. Similar to the leg press testing, training consisted of a slow and controlled eccentric phase (from near ~ 180° angle in the knee joint to ~ 90° knee flexion), a short stop (< 1 s) at the bottom of the movement, before the concentric phase back up to near ~ 180° knee angle. The execution of each repetition ended with a plantar flexion, which has previously been shown to induce neural adaptations in the lower leg (Fimland et al. 2009; Toien et al. 2018a). In accordance with previous research, emphasis was placed on performing the concentric phase as forcefully and rapidly as possible (i.e., maximal intended velocity) (Toien et al. 2018a) and 3-min of rest were used between each set.

### Statistical analyses

IBM SPSS statistics software version 23 (Chicago, IL, USA) was used for statistical analyses, and GraphPad Prism 6 (San Diego, CA, USA) was used for graphic illustrations. The data were assessed for normality with Q-Q plots and Shapiro–Wilk’s test for normality. All variables exhibited a normal distribution, and as such parametric tests were applied. First, to detect within-group changes from pre- to post-test a paired samples *t* test was applied to each dependent variable (i.e., weight, 1RM, RFD, evoked potentials recordings, and SERCA-expression). Second, the between-group difference for the changes from pre- to post-test was studied using analysis of variance (ANOVA). Data are presented as mean ± standard deviation (SD) in the tables and with 95% confidence intervals of the mean difference in brackets in text, and as mean ± standard error (SE) and individual values in figures. *p* ≤ 0.05 was considered statistically significant.

## Results

### Subjects and adherence

All subjects completed the study, and the MST group completed 23 ± 2 training sessions. Body mass did not change throughout the study (MST pre: 77.2 ± 7.9 kg, post: 77.5 ± 8.3 kg; [− 1.1 to 1.8 kg]; *p* = 0.566; CG pre: 82.2 ± 7.6 kg, post: 81.8 ± 7.0 kg; [− 2.1 to 1.3 kg]; *p* = 0.599). One result from CG was excluded from V-wave-analysis, as the participant was unable to maximally contract during V-wave trials at post-test (< 90% MVC in all trials).

### Maximal strength and rate of force development

**Fig. 1:**
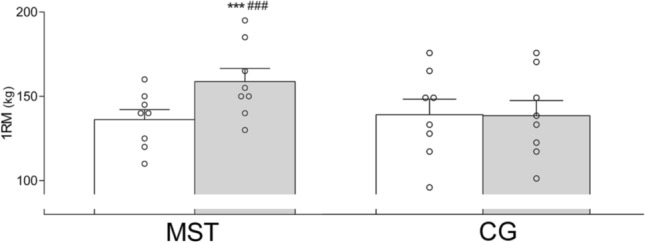
Leg press one repetition maximum (1RM) before (white bar) and after (grey bar) the training intervention in the maximal strength training (MST) group and control group (CG). ***p ≤ 0.001 different from pre-test within group, ^###^*p* ≤ 0.001 different between groups from pre- to post-test. Data are presented as mean ± SE and individual responses

**Fig. 2:**
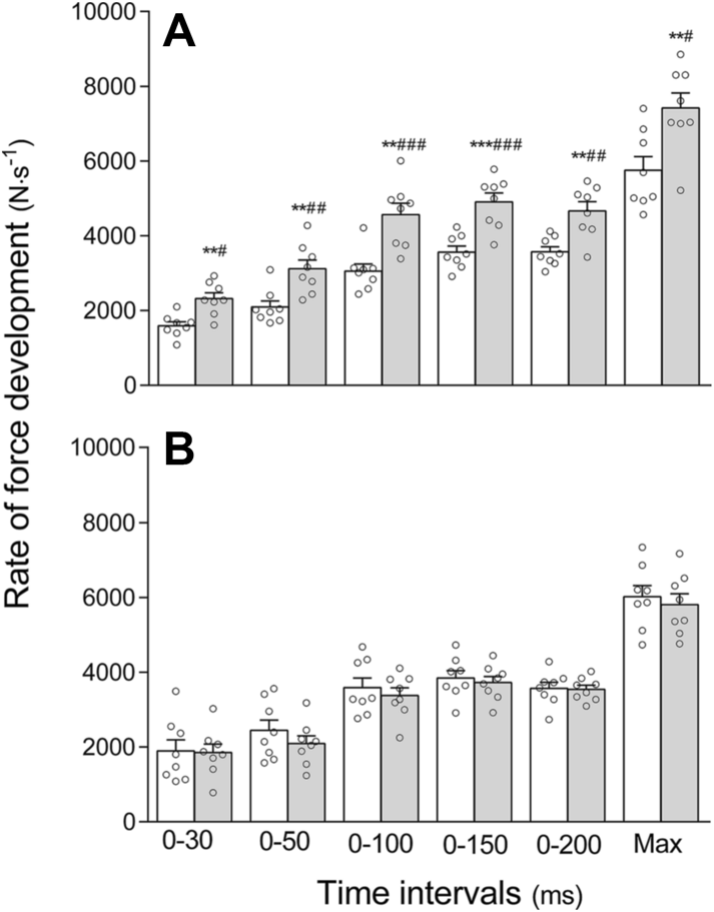
Rate of force development in time intervals 0–30 ms, 0–50 ms, 0–100 ms, 0–150 ms, 0–200 ms, and in the steepest 10 ms of the force–time curve (max) before (white bar) and after (grey bar) the training intervention in the **A** maximal strength training (MST) group and **B** control group (CG). **p ≤ 0.01; ***p ≤ 0.001 different from pre-test within group, ^#^*p* ≤ 0.05; ^##^*p* ≤ 0.01; ^###^*p* ≤ 0.001 different between groups from pre- to post-test. Data are presented as mean ± SE and individual responses

Leg press 1RM increased following strength training with 17 ± 9% (136 ± 17 to 159 ± 22 kg; [13 to 32 kg]; *p* < 0.001; Fig. 1), which was significantly different from the CG ([14 to 33 kg]; *p* < 0.001). No pre- to post-test difference was detected in CG (136 ± 24 to 135 ± 24 kg; [− 5 to 4 kg]; *p* = 0.732). RFD increased from pre- to post MST with 49 ± 36% (0–30 ms; [359 to 1100 N s^−1^]; *p* = 0.001), 53 ± 41% (0–50 ms; [440 to 1606 N s^−1^]; *p* = 0.002), 52 ± 34% (0–100 ms; [748 to 2273 N s^−1^]; *p* = 0.001), 39 ± 24% (0–150 ms; [755 to 1923 N s^−1^]; *p* < 0.001), 31 ± 21% (0–200 ms; [541 to 1645 N s^−1^]; *p* = 0.001) and 32 ± 22% (maximal RFD; [788 to 2549 N s^−1^]; *p* = 0.002). The time intervals are presented in Fig. 2. No within-group difference was detected in any time interval in CG (all *p* > 0.05). There was a training-induced between-group difference in all time intervals (all *p* < 0.05).

### Evoked reflex recordings

**Fig. 3:**
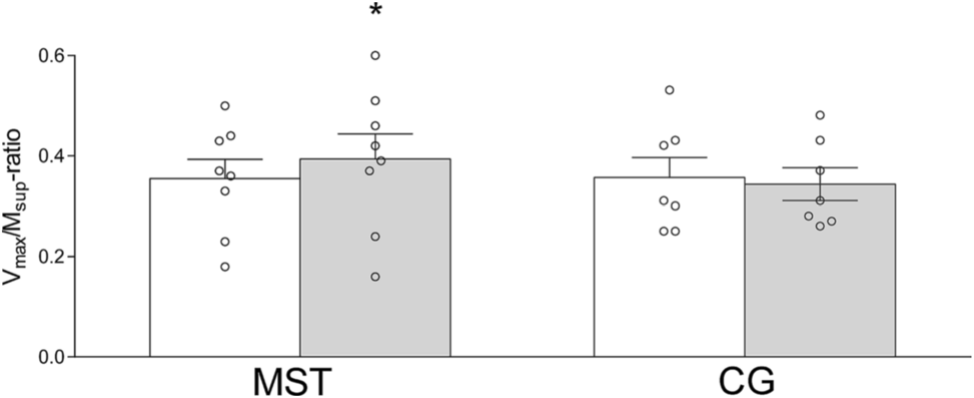
Soleus *V*_max_/*M*_sup_-ratio before (white bar) and after (grey bar) the training intervention in the maximal strength training (MST) group and control group (CG). *V*_max_/*M*_sup_-ratio; maximal V-wave amplitude/maximal M-wave amplitude. *p ≤ 0.05 different from pre-test within group. Data are presented as mean ± SE and individual responses

MST increased *V*_max_/*M*_sup_-ratio with 9 ± 14% (0.36 ± 0.11 to 0.39 ± 0.14; [− 0.01 to 0.09]; *p* = 0.046, Fig. 3). No within-group difference was detected in CG (*p* = 0.734). The strength training-induced increase in *V*_max_/*M*_sup_-ratio displayed a tendency to be different from the CG ([− 0.03 to 0.13]; *p* = 0.093). Soleus *H*_max_/*M*_max_-ratio remained unchanged following the training period in MST ([− 0.19 to 0.06]; *p* = 0.268; Table 1) and the CG ([− 0.10 to 0.12]; *p* = 0.812). No pre- to post-test difference was observed in absolute evoked amplitudes in either group (Table 1).

### Relative mRNA and muscle protein expressions

**Fig. 4:**
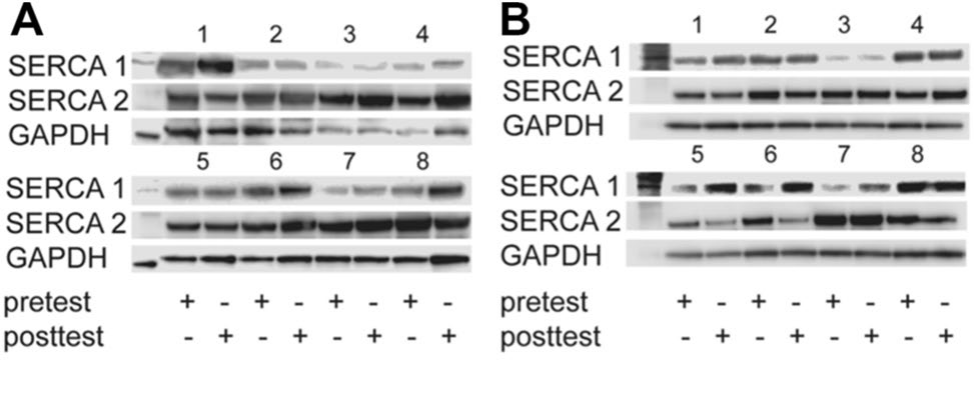
Immunoblot with pre- and post-test expression of SERCA1 (~ 110 kDa), SERCA2 (~ 110 kDa) and GAPDH (~ 37 kDa) in muscle biopsies from participants 1–8 in **A** maximal strength training group and **B** control group

The relative mRNA expression of SERCA1a (MST: 0.12 ± 0.03 to 0.09 ± 0.02; [− 0.06 to 0.01]; *p* = 0.140; CG: 0.07 ± 0.02 to 0.07 ± 0.02; [− 0.03 to 0.02]; *p* = 0.903) and SERCA2a (MST: 2.76 ± 2.29 to 2.20 ± 0.97; [− 2.34 to 1.21]; *p* = 0.478; CG: 2.02 ± 1.20 to 2.42 ± 1.15; [− 0.76 to 1.57]; *p* = 0.438) was unchanged in both groups. The relative protein expression of SERCA1 (1.00 ± 0.71 to 1.69 ± 1.37; [− 0.50 to 1.87]; *p* = 0.214) and SERCA2 (1.00 ± 0.48 to 1.58 ± 0.93; ([− 0.52 to 1.68]; *p* = 0.253) remained unchanged following MST. Similarly, no change was detected in the CG in SERCA 1 (1.00 ± 0.39 to 1.38 ± 0.60; [− 0.34 to 1.10]; *p* = 0.257) or SERCA 2 (1.00 ± 0.31 to 0.84 ± 0.40; [− 0.49 to 0.18]; *p* = 0.303) (Fig. 4).

## Discussion

To better understand whether the well-established efferent neural drive adaptations may be accompanied by adaptations in key muscular factors involved in calcium-dependent contractility following high intensity strength training, we investigated efferent neural drive and muscular SERCA-adaptations before and after 8 weeks of MST. As expected, maximal strength and RFD increased in the healthy, young male participants. This improvement in strength was accompanied by enhanced efferent neural drive, measured as increased *V*_max_/*M*_sup_-ratio in the soleus muscle, thus confirming the first part of our hypothesis. However, the relative protein expression and mRNA expression of SERCA obtained from vastus lateralis biopsies did not change following MST, thus we reject the second part of our hypothesis. These results confirm that efferent neural drive adaptations are a major contributor to MST-enhanced force production. In contrast, SERCA expression, a key muscular factor in active Ca^2+^ pumping, did not contribute to enhanced force production in the present study.

### Maximal strength training, force production and efferent neural drive

The increase in 1RM and RFD was similar to previous results from our laboratory using MST in multi-joint exercises in the lower extremities in young, healthy individuals (Ovretveit and Toien 2018; Toien et al. 2018a). Of note, supervised MST with its high concentric intensity along with a slow and controlled eccentric phase is considered to be safe and effective in a range of populations (Mosti et al. 2013; Wang et al. 2017; Hoff et al. 1999, 2007; Toien et al. 2018a). The improvements in maximal strength and RFD were accompanied by increased efferent neural drive (*V*_max_/*M*_sup_-ratio). Interestingly, we have recently documented, albeit in an elderly population, that a potential learning effect of training is not manifested as increased *V*_max_/*M*_sup_-ratio (Unhjem et al. 2021). This strengthens the assumption with which we can assume that the increases observed in *V*_max_/*M*_sup_-ratio are an effect of the strength training itself, although we cannot rule out that a learning effect contributed to the increases in 1RM and RFD. *V*_max_/*M*_sup_-ratio is thought to reflect maximal firing frequency and motor unit recruitment to maximally contracting skeletal muscle (Aagaard et al. 2002b; Vila-Chã et al. 2012). A strong relationship between RFD and efferent neural drive has previously been evident (Aagaard et al. 2002a), and firing frequency in particular appears to be a major determinant for rapid force production (Van Cutsem et al. 1998). This is especially true in the early phase of contraction (de Ruiter et al. 2004). Thus, it is interesting that early-phase RFD has not consistently increased following strength training (Andersen et al. 2010), which is in contrast to the present study. However, this discrepancy may be due to the use of maximal intended velocity in the concentric phase in the present study, where maximal firing frequency from the start of contraction is emphasized (Hoff et al. 2007). The increase in early phase RFD in the present study, taken together with the notion that motor unit recruitment is typically near maximal in young, healthy individuals (Goodall et al. 2009; Bigland-Ritchie et al. 1992), makes it likely that the increased *V*_max_/*M*_sup_-ratio was predominantly due to increased firing frequency.

An increase in *V*_max_/*M*_sup_-ratio along with an unchanged resting or low contraction *H*_max_/*M*_max_-ratio following strength training is commonly reported in the literature (Aagaard et al. 2002b; Vila-Chã et al. 2012; Toien et al. 2018a; Duclay and Martin 2005). The V-wave is an electrophysiological variant of the H-reflex, and thus these two reflexes have commonly been used together to more accurately determine the cite of adaptation. The H-reflex is thought to reflect the excitability of the α-motoneurons along with pre/post-synaptic inhibition (Aagaard et al. 2002b), whereas the V-wave also includes descending neural drive to induce a maximal muscle contraction and is determined by antidromic clearing (Aagaard et al. 2002b). The unchanged *H*_max_/*M*_max_-ratio in the present study along with an increased *V*_max_/*M*_sup_-ratio, therefore, indicates that the increased efferent neural drive is largely dependent on corticospinal factors. Indeed, since the *V*_max_/*M*_sup_-ratio was measured in the soleus muscle in the current study, which is not a prime mover in the leg press training, this underpins that adaptations in efferent neural drive may be a central adaptation that is detectable in several muscle groups. In support of this notion, we have recently documented, albeit in an elderly population, that *V*_max_/*M*_sup_-ratio also increased in an untrained limb following MST of the contralateral limb (Toien et al. 2018b). Still, contrasting the *V*_max_/*M*_sup_-ratio to the *H*_max_/*M*_max_-ratio should be made with caution, since the two reflexes are measured under different conditions, i.e., low force and maximal muscle contraction, and thus may reflect two distinct adaptations in the nervous system.

### Maximal strength training and relative expression of SERCA

Given the substantial MST-induced increase in maximal strength and RFD, and especially considering the well-documented improvements in skeletal muscle efficiency (Berg et al. 2018; Hoff et al. 1999, 2002; Storen et al. 2008), even in five well-trained cyclists (Barrett-O’Keefe et al. 2012), it is somewhat surprising that the enhanced efferent neural drive was not accompanied by changes in relative mRNA expression and protein expression of SERCA1 and SERCA2. However, it should be noted that these measurements were obtained from different muscle groups, and do not necessarily accurately reflect each other. However, since muscle biopsies were taken from the vastus lateralis of the thigh, a prime mover in the leg press exercise, we feel confident that any potential change in SERCA expression in this muscle could be detected. Thus, it appears that the relative density of energy-dependent Ca^2+^-pumping proteins, a major contributor to the energy cost of muscle contractions (Barclay et al. 2007), may not contribute to the effects observed following MST in the current or previous investigations. However, the lack of SERCA alterations in the skeletal muscle bed coupled with enhanced force production after MST may contribute to reduce energy-requirements in low-force contractions, by allowing more energy-efficient and fatigue resistant type I fibers potentially to perform the work previously carried out by less energy-efficient and fatigue resistant type II fibers. As the rise in force production is more energy dependent than force maintenance (Russ et al. 2002), the MST-induced increase in RFD may also contribute to less involvement of Ca^2+^-pumping proteins because of a shorter force development phase.

Our results are in contrast to the increased relative protein expression of SERCA1 and SERCA2 previously reported following 5 weeks of sprint training Ortenblad et al. (2000). However, despite that sprint training is performed with short bursts of explosive movements, there are important distinctions separating it from high intensity strength training which may explain the contrasting results. While sprint training is typically performed with no external resistance, relatively short recovery time and emphasis on enhanced anaerobic energy turnover, MST is performed with heavy external resistance and longer recovery time to emphasise maximal performance in each lift. These differences could explain the different effects on relative SERCA expression in the Ortenblad et al. (2000) study and present study.

Of importance, in younger individuals, there appears to be a more prominent effect on relative SERCA expression following endurance training, where a reduction has been reported (Green et al. 2003, 2011; Majerczak et al. 2008; Skovgaard et al. 2014). This indicates that it may rather be prolonged exercise with low or moderate-force muscle contractions that causes adaptations in relative SERCA expression and not muscle contractions with very high concentric force and maximal efferent neural drive. Thus, a reduced relative SERCA expression may be an advantageous strategy for energy reduction to avoid fatigue.

Some of the differences in the present study compared to previous literature may be explained by differences in biopsy sampling time. However, this is difficult to examine, since previous studies have not always reported the sampling time relative to the first test session, i.e., whether physical testing was performed before or after the biopsy was obtained (Ortenblad et al. 2000; Green et al. 2003; Majerczak et al. 2008). Sampling time at post-test has been similar to what was used in the present study, typically ranging from 2 to 4 days between the last training session and muscle biopsy (Ortenblad et al. 2000; Green et al. 2003, 2011; Majerczak et al. 2008; Skovgaard et al. 2014). Thus, we cannot rule out that differences in stimulus prior to sampling of the biopsies may have influenced the conflicting results, although this is likely not the main explanation.

### Limitations

We should address some limitations in the present study. It is always preferable with a high number of subjects and a long intervention period in this type of study. However, due to the invasive nature of muscle biopsies and somewhat painful measurements using electrical stimulation, we sought to minimise the number of participants. Considering the number of participants used in similar studies previously (Green et al. 2003, 2011; Ortenblad et al. 2000), along with the successful detection of training-induced increases in 1RM, RFD and *V*_max_/*M*_sup_-ratio, the number of participants and length of the intervention appeared sufficient in the present study. More variables related to the muscle biopsy samples along with anthropometric measurements of muscle volume and muscle mass would also contribute to a more thorough understanding of muscular adaptations resulting from MST. However, previous studies from our lab, where MST has been applied have reported no change in muscle mass or muscle volume following 8 weeks of training (Berg et al. 2018; Heggelund et al. 2013). Moreover, obtaining all measurements from the same muscle would have been preferable. However, an inherent limitation with the H-reflex and V-wave method is that it is very challenging to perform in the thigh musculature, and as such is commonly measured in the calf muscles because of its more distal location from the spine (Aagaard et al. 2002b; Vila-Chã et al. 2012). Since this method has the advantage of allowing for measurements under maximal muscle contraction and maximal electrical stimulation, which are considered appropriate conditions to obtain neural measurements following strength training (McNeil et al. 2013), this method was chosen in the present study. Importantly, previous studies from our laboratory has shown that leg press strength training, where a plantar flexion is used to finish the movement has successfully induced changes in *V*_max_/*M*_sup_-ratio measured in the soleus muscle (Fimland et al. 2009; Toien et al. 2018a), indicating alterations in the central motor pathway following strength training, that are detectable even in muscle groups not preferentially targeted in the training intervention.

## Conclusion

Leg press MST performed with heavy loads and maximal mobilization of force in the concentric phase increases maximal strength and dynamic RFD in young, healthy males with concomitant increases in efferent neural drive. No changes were observed in relative mRNA and relative protein expression of SERCA from vastus lateralis biopsies, which appears to be unaffected following 8 weeks of MST in younger adults.

## Data Availability

N/A

## Abbreviations

ANOVA: Analysis of variance
cDNA: Complementary DNA
CG: Control group
EMG: Electromyography
*H*_max_: Maximal H-reflex during 10% MVC
ICC: Intraclass correlation coefficient
*M*_max_: Maximal M-wave during 10% MVC
mRNA: Messenger ribonucleic acid
MST: Maximal strength training
*M*_sup_: Maximal M-wave during MVC
MVC: Maximal voluntary contraction
RFD: Rate of force development
RM: Repetition maximum
RNA: Ribonucleic acid
SERCA: Sarcoplasmic reticulum Ca^2^^+^ ATPase
SD: Standard deviation
SE: Standard error
SR: Sarcoplasmic reticulum
*V*_max_: Maximal V-wave

## Code availability

N/A
